# Case report: Disease phenotype associated with simultaneous biallelic mutations in *ABCA4* and *USH2A* due to uniparental disomy of chromosome 1

**DOI:** 10.3389/fgene.2022.949437

**Published:** 2022-08-16

**Authors:** R. Villafuerte-De la Cruz, O. F. Chacon-Camacho, A. C. Rodriguez-Martinez, N. Xilotl-De Jesus, R. Arce-Gonzalez, C. Rodriguez-De la Torre, J. E. Valdez-Garcia, A. Rojas-Martinez, J. C. Zenteno

**Affiliations:** ^1^ Tecnologico de Monterrey, Escuela de Medicina y Ciencias de la Salud, Monterrey, Mexico; ^2^ Carrera Médico Cirujano, Facultad de Estudios Superiores Iztacala, Universidad Nacional Autónoma de México, Mexico City, Mexico; ^3^ Genetics Department, Institute of Ophthalmology “Conde de Valenciana”, Mexico City, Mexico; ^4^ Department of Ophthalmology, University Hospital and Faculty of Medicine, Autonomous University of Nuevo Leon (UANL), Monterrey, Mexico; ^5^ Institute for Obesity Research, Tecnologico de Monterrey, Escuela de Medicina y Ciencias de la Salud, Monterrey, Mexico; ^6^ Biochemistry Department, Faculty of Medicine, National Autonomous University of Mexico (UNAM), Mexico City, Mexico

**Keywords:** uniparental disomy, retinal dystrophy, *USH2A* gene, *ABCA4* gene, Stargardt disease, Usher syndrome

## Abstract

Inherited retinal diseases (IRDs) represent a spectrum of clinically and genetically heterogeneous disorders. Our study describes an IRD patient carrying *ABCA4* and *USH2A* pathogenic biallelic mutations as a result of paternal uniparental disomy (UPD) in chromosome 1. The proband is a 9-year-old girl born from non-consanguineous parents. Both parents were asymptomatic and denied family history of ocular disease. Clinical history and ophthalmologic examination of the proband were consistent with Stargardt disease. Whispered voice testing disclosed moderate hearing loss. Next-generation sequencing and Sanger sequencing identified pathogenic variants in *ABCA4* (c.4926C>G and c.5044_5058del) and *USH2A* (c.2276G>T). All variants were present homozygously in DNA from the proband and heterozygously in DNA from the father. No variants were found in maternal DNA. Further analysis of single nucleotide polymorphisms confirmed paternal UPD of chromosome 1. This is the first known patient with confirmed UPD for two recessively mutated IRD genes. Our study expands on the genetic heterogeneity of IRDs and highlights the importance of UPD as a mechanism of autosomal recessive disease in non-consanguineous parents. Moreover, a long-term follow-up is essential for the identification of retinal features that may develop as a result of *USH2A*-related conditions.

## Introduction

Inherited retinal dystrophies (IRDs) are a group of phenotypically and genetically heterogeneous diseases affecting up to 4.5 million people worldwide and leading to disabling impairment of vision in affected individuals (Hohman, 2017). IRDs can be classified according to its progression (progressive or stationary), the presence or absence of concurrent extraocular anomalies (syndromic or non-syndromic), and the predominantly affected photoreceptor cell (rod dystrophy, cone dystrophy, or generalized dystrophies) (Berger et al., 2010). Recent advances in molecular genetic diagnostic techniques, such as next-generation sequencing (NGS), have led to the identification of the underlying genetic cause in a majority of IRD patients (Chiang et al., 2015; Bernardis et al., 2016). In addition, molecular diagnosis through NGS has allowed the recognition of the ethnic-specific mutational spectrum in IRDs (Avela et al., 2018; Zenteno et al., 2020) and has unmasked complex genotypes which were previously associated with diagnostic odysseys (Jones et al., 2017; González-Del Pozo et al., 2020).

*ABCA4* and *USH2A* are two of the most frequently mutated genes in IRD patients worldwide. Biallelic mutations in *ABCA4*, a gene located at 1p22.1 and encoding the retina-specific transmembrane protein involved in the transport of retinoids in the visual cycle, cause Stargardt disease (STGD), a recessive macular dystrophy regarded as one of the most prevalent inherited retinal disorders in humans (Tsang and Sharma, 2018). To date, about 1700 different pathogenic variants in *ABCA4* have been identified in STGD and other related retinal phenotypes collectively known as *ABCA4*-associated retinopathies (Stenson et al., 2020). Most disease-causing *ABCA4* variants are single-nucleotide substitutions that predict missense changes in the protein. On the other hand, biallelic mutations in *USH2A* gene (located at 1q41), which encodes for usherin, a basement membrane protein in the inner ear and retina, underlie ∼20% of cases of non-syndromic retinitis pigmentosa, ∼7% of non-syndromic hearing loss, and up to 80% of cases of Usher syndrome (USH) type II (Koenekoop et al., 1999; McGee et al., 2010; Del Castillo et al., 2022). USH is an autosomal recessive disorder characterized by visual loss due to retinitis pigmentosa, sensorineural hearing impairment, and variable vestibular dysfunction. Around 1800 different *USH2A* pathogenic variants have been described to date (Stenson et al., 2020), including nonsense and missense mutations, splicing variants, small deletions and insertions, small indels, and large rearrangements (Gao et al., 2021).

Uniparental disomy (UPD), defined as the presence in a diploid genome of a chromosome pair derived from one progenitor, is a rare cause of recessively inherited disorders (Yamazawa et al., 2010; Erger et al., 2018). To the best of our knowledge, only 2 STGD and 3 Usher syndrome cases have been demonstrated to occur due to chromosome 1 UPD (Rivolta et al., 2002; Fingert et al., 2006; Riveiro-Alvarez et al., 2007; Fu et al., 2020; Wang et al., 2021).

In this work, we describe the clinical and molecular findings in a patient carrying simultaneous *ABCA4* and *USH2A* biallelic mutations as a result of paternal UPD of chromosome 1. To the best of our knowledge, this is the first known patient with confirmed UPD for two recessively mutated IRD genes.

## Material and methods

### Clinical examination

The protocol was approved by the Institutional Review Board of the Institute of Ophthalmology “Conde de Valenciana”, Mexico City. All procedures followed the tenets of the Helsinki Declaration, and the patient’s parents gave written consent for their inclusion in the study. The participating proband underwent a detailed clinical history and a full ophthalmological evaluation. Additionally, color fundus photography (CFP), fundus autofluorescence (FAF) imaging (Zeiss Visucam NM, Carl Zeiss, Germany), spectral domain optical coherence tomography (Cirrus HD-OCT, Carl Zeiss Meditec AG, Jena, Germany), and electrophysiological assessment (full-field and multifocal electroretinography) were performed. Parents were asymptomatic and consented to donate peripheral blood for DNA analyses.

### DNA isolation and next-generation sequencing

Genomic DNA (gDNA) was extracted from peripheral blood leukocytes using the QIAamp DNA Blood kit (QIAGEN, Hilden, Germany). gDNA quantification and purity of samples were measured using a NanoDrop 2000 spectrophotometer (Thermo Fisher Scientific, Waltham, MA). All exon regions of the Invitae Inherited Retinal Disorders Panel (293 genes) (Invitae, San Francisco, CA) were sequenced. Briefly, gDNA was enriched for target regions using a hybridization-based protocol and sequenced using Illumina technology (Illumina, San Diego, CA). All targeted regions were sequenced with ≥ ×50 depth or were supplemented with additional analysis. Reads were aligned to a reference sequence GRCh37/19 human reference genome, and sequenced changes were identified and interpreted in the context of a single clinical relevant transcript, indicated as follows. Enrichment and analysis focus on the coding sequence of the indicated transcripts, 20 bp of flanking intronic regions, and other specific genomic regions demonstrated to be causative of disease at the time of the assay design. This assay achieves >99% analytical sensitivity and specificity for single-nucleotide variants, and insertions and deletions <15 bp in length. Designation of pathogenic/likely pathogenic variants was carried out following the American College of Genetics and Genomic (ACMG) guidelines (Richards et al., 2015). Exonic deletions and duplications (CNVs) were called using an in-house algorithm (Invitae) that determines the copy number at each target by comparing the read depth for each target in the proband sequence with both mean read depth distributions, obtained from a set of clinical samples.

### Primer design and PCR

For PCR amplification of *ABCA4* and *USH2A* exons 35-36 and 13, respectively, each 25 μL reaction contained 1X buffer, 200 ng of genomic DNA, 0.2 mM of each deoxynucleotide triphosphate, 2U Taq polymerase, 1 mM of forward and reverse primers, and 1.5 mM of MgCl2. The primers are the following: for *ABCA4*; 35F forward 5′-GCA​GCG​TCT​CAG​ATG​TCC​TC-3′ and 35R reverse 3′-CGG​TGG​TGA​GAA​TCC​TCT​CA-5′; 36F forward 5′-GTA​TCT​TCT​CCT​CCT​TCT​GC-3′ and 36R reverse 3′-ACA​CAC​AAG​CTC​CAC​CTT​G-5′; and for *USH2A*, 13F forward 5′-GCA​GTA​GCA​TTG​TTT​GTG​TCT​C-3′ and 13R reverse 3′-ATT​TGT​AGA​AGC​CAC​AAA​CC-5'. The PCR temperature program included 30 cycles of denaturation at 97°C for 1 min, annealing at 60°C for 1 min, and extension at 72°C for 1 min. Sanger sequencing was performed with the BrilliantDye Terminator v1.1 Cycle Sequencing Kit (NimaGen BV, Netherlands), adding about 15 ng of template DNA, 3.5 ml of the 5X sequencing buffer, 1 ml BrilliantDye v1.1 rr premix, 1 ml primer, and 13.5 ml water in each reaction (20 ml total) and using a temperature program that included 25 cycles of denaturation at 96°C for 10 s, annealing at 50°C for 5 s, and extension at 60°C for 4 min. Samples were analyzed in a Spectrum Compact CE System (Promega Corporation, WI, United States).

### Chromosome 1 SNP analysis

Variant call format (VCF) files obtained from gene panel sequencing (Invitae) and containing the full list of variants identified (including pathogenic, likely pathogenic, VUS, likely benign, and benign variants) were analyzed. Subsequently, variants occurring in genes located at chromosome 1 were manually selected and verified for their zygosity status. Only variants sequenced with ≥50 depth were included in the SNP analysis.

### Sanger sequencing of *ABCA4* and *USH2A* genes

Specific oligonucleotide primers were designed by amplification of exons 35 and 36 of *ABCA4* gene (NM_000350.3), and exon 13 of *USH2A* (NM_206933.4). Sanger nucleotide analysis was performed by BigDye Terminator v1.1 chemistry on a Spectrum Compact CE System (Promega Corporation, WI). Confirmatory Sanger sequencing of the pathogenic variants was carried out in DNAs from the proband and her parents.

## Results

### Clinical features

The index case is a 9-year-old Mexican girl who presented with a 6-year history of uncorrected visual impairment and right hypertropia. This was accompanied by light sensitivity and dark-to-light adaptation difficulties. Her past ocular history was remarkable for occlusion therapy from age 3–6 years. She was the product of the first pregnancy of non-consanguineous parents. Family history disclosed three miscarriages and one instance of stillbirth in her otherwise healthy mother. Her parents denied a personal or family history of ocular diseases.

**FIGURE 1:**
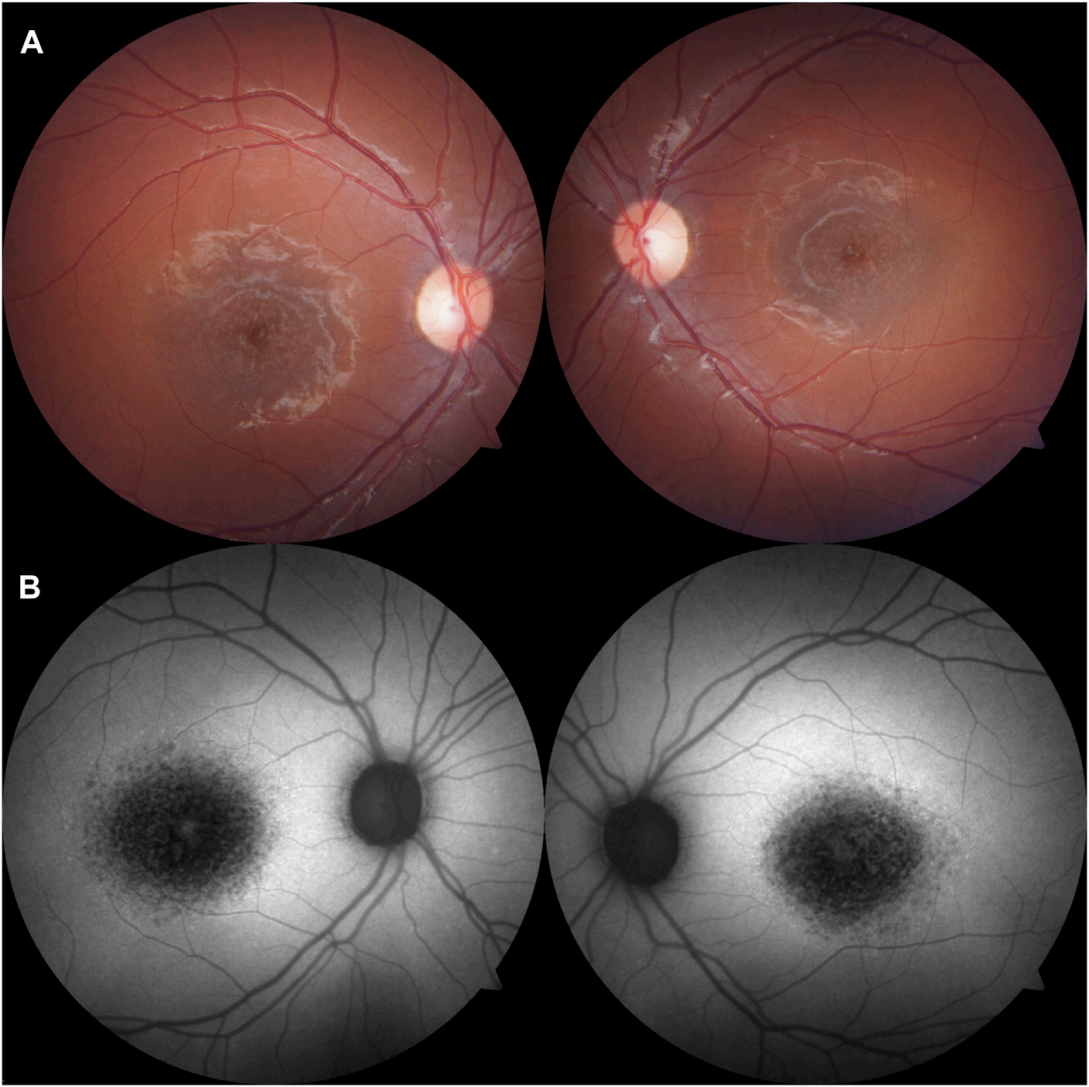
**(A)** Color fundus photography revealed a macular bull’s eye appearance with small pisciform yellow-white flecks scattered within the posterior pole on both eyes. **(B)** Fundus autofluorescence (FAF) revealed a central area of macular hypoautofluorescence along with retinal flecks extending centrifugally and appearing hypoautofluorescent in the center and hyperautofluorescent in the periphery. The peripapillary sparing retina should be noted, as demonstrated by fundus autofluorescence.

**FIGURE 2:**
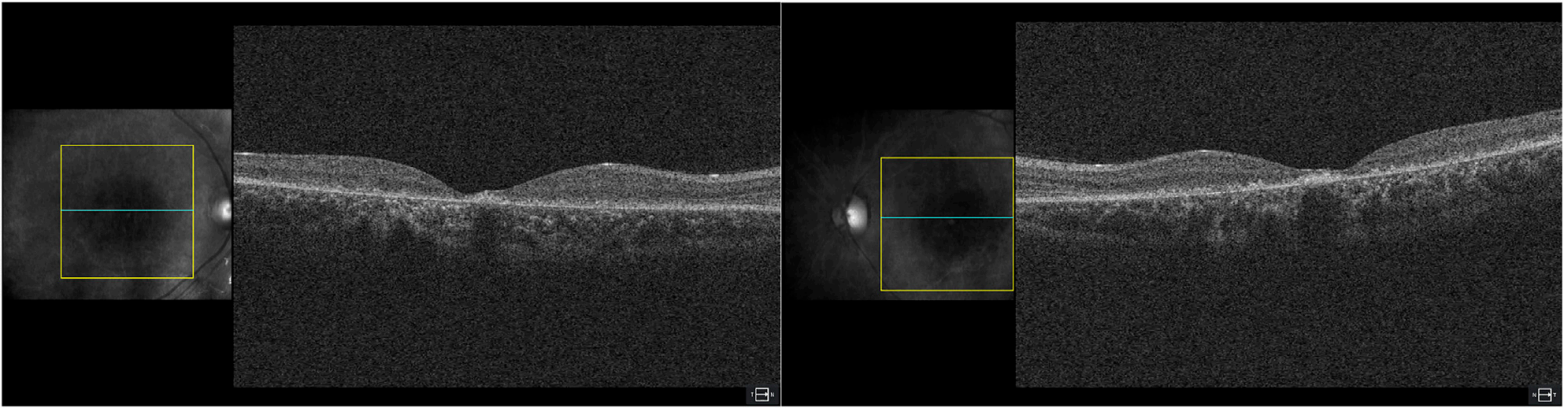
High-resolution spectral domain optical coherence tomography (SD-OCT) demonstrated decreased retinal thickness, particularly at the foveola, with loss of inner and outer photoreceptor segment layers and hyper-reflective deposits below the RPE corresponding to flecks.

**FIGURE 3:**
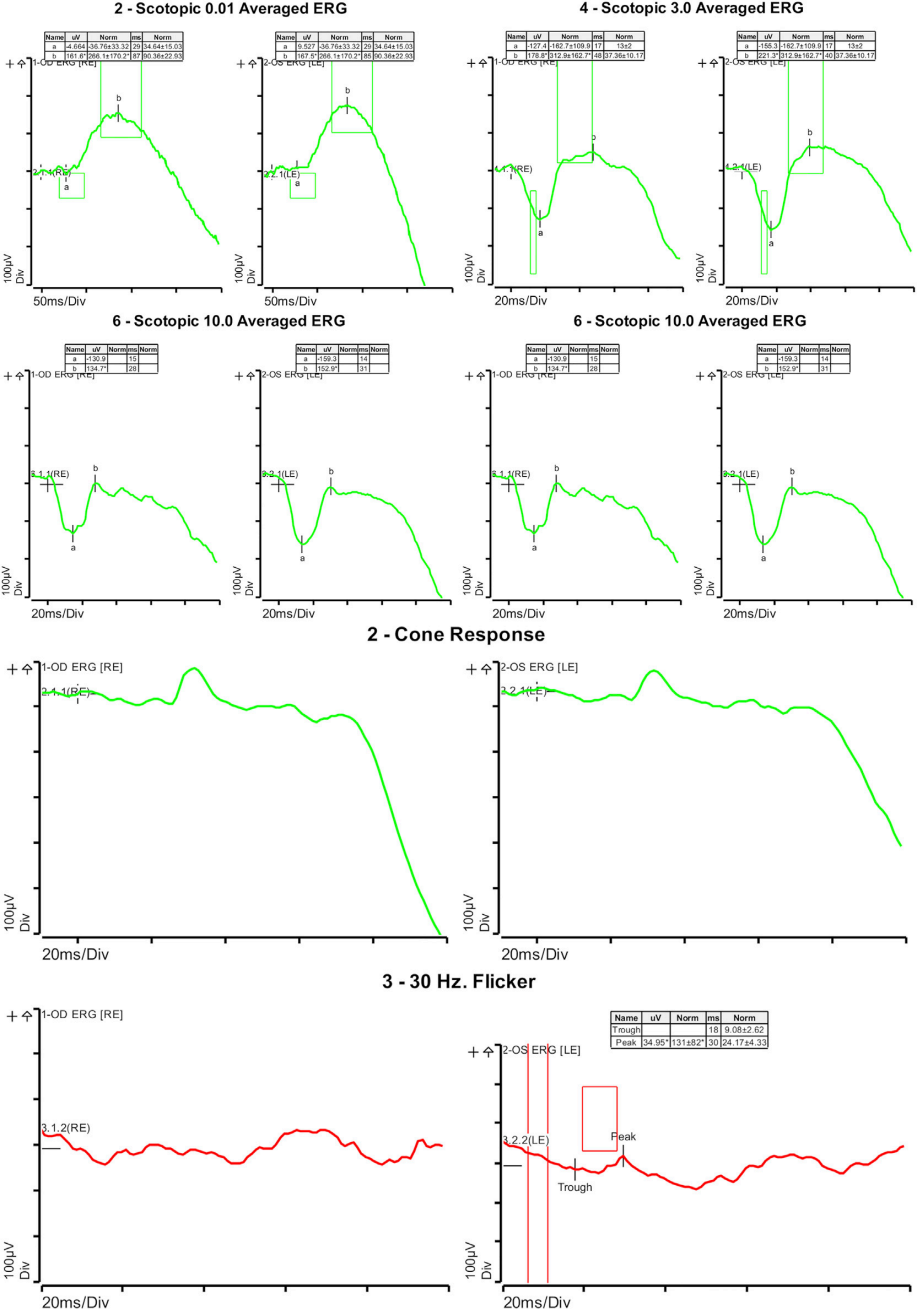
Electroretinogram with subnormal rod response and severe reduction of cone response.

On ophthalmologic examination, best corrected visual acuity on the right eye was 20/200 (1.00 logMAR) and left eye was 20/160 (0.90 logMAR). The right pupillary diameter was 3 mm, while the left pupil diameter was 1.5 mm. There was no afferent pupillary defect, and both pupils were reactive to light and accommodation. Orthoptic assessment showed right hypertropia, and her extraocular movements were within normal limits. The color vision assessment, evaluated with the Ishihara test, was unremarkable. No anomalies were identified in the anterior segment at slit lamp examination. Funduscopic examination revealed a bilateral vermillion appearance. No evidence of bone spicule pigmentation was observed. Color fundus photography revealed a macular bull’s eye appearance with small pisciform yellow-white flecks scattered within the posterior pole (Figure 1A). Fundus autofluorescence (FAF) revealed a central area of macular hypoautofluorescence along with retinal flecks extending centrifugally and appearing hypoautofluorescent in the center and hyperautofluorescent in the periphery. The peripapillary sparing retina should be noted, as demonstrated by fundus autofluorescence (Figure 1B). High-resolution spectral domain optical coherence tomography (SD-OCT) demonstrated decreased retinal thickness, particularly at the foveola with the loss of inner and outer photoreceptor segment layers and hyper-reflective deposits below the RPE corresponding to flecks (Figure 2). Electroretinogram records show subnormal rod and cone responses with severely reduced amplitude and implicit time, especially evident in flicker response (Figure 3). General audiological examination using the patient’s responses to whispered questions and the general hearing test revealed moderate hearing loss, with no speech impairment. Parents refused formal audiological tests to be performed in the proband. Retinal phenotypic features and electrophysiological studies in our patient were consistent with STGD.

### Molecular analysis

**FIGURE 4:**
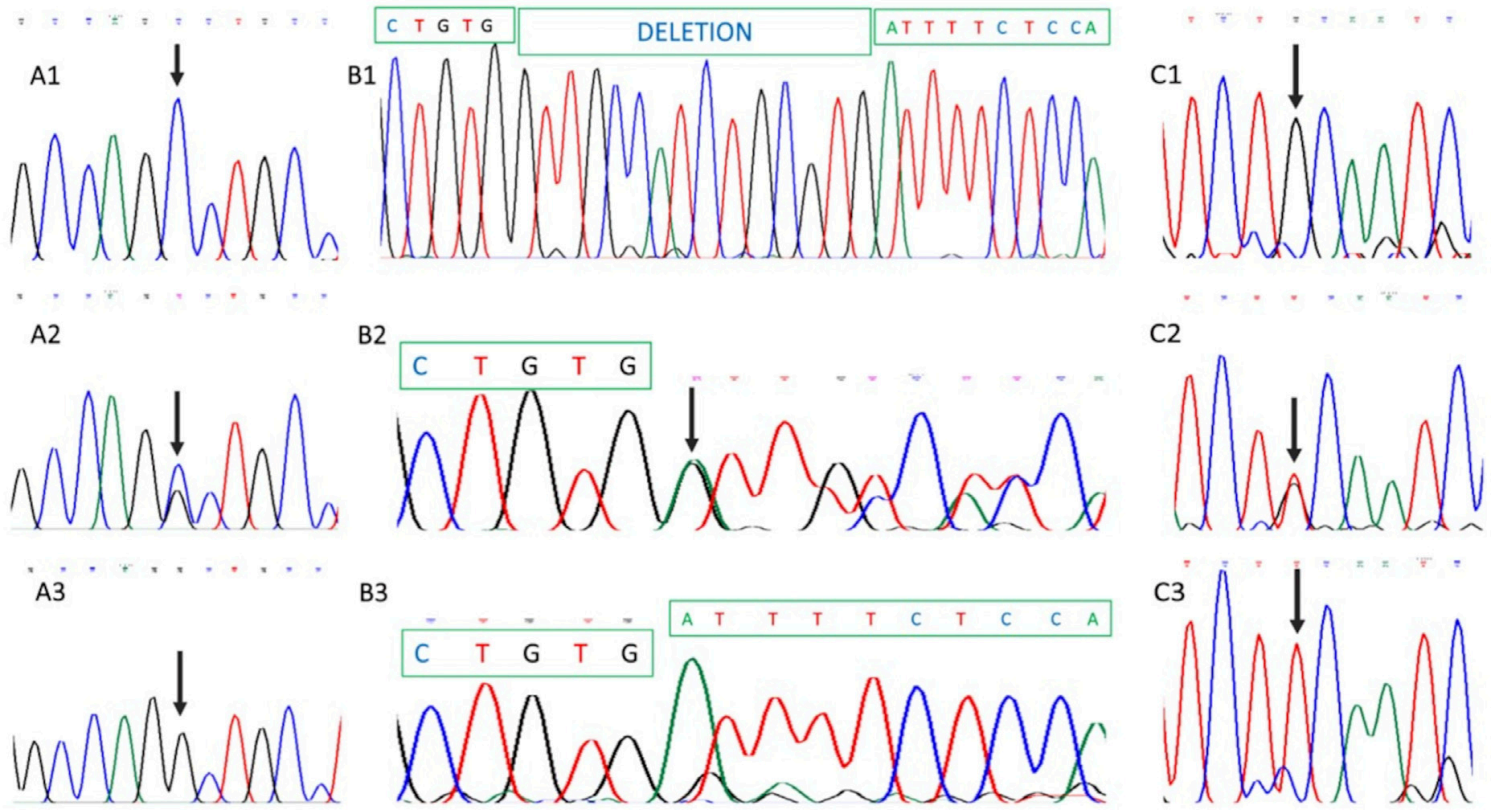
Partial DNA Sanger sequencing of *ABCA*4 and *USH2A* genes. **(A1)**, **(B1)**, and **(C1)** show partial DNA sequences from the healthy mother. **(A1)** and **(A2)** correspond to *ABCA4* partial sequences, while **(C1)** shows the *USH2A* sequence. As shown, no pathogenic variants were identified in maternal DNA. **(A2)**, **(B2),** and **(C2)** show paternal DNA analysis demonstrating heterozygosity for *ABCA4* and *USH2A* pathogenic variants. **(A3)**, **(B3),** and **(C3)** show homozygosity for *ABCA4* **(A3** and **B3)** and *USH2A* **(C3)** mutations in proband’s DNA. Involved nucleotides are arrowed.

Sequencing of a panel of 293 retinal dystrophy genes in DNA from the index case identified homozygosity for a pathogenic *ABCA4* complex allele and homozygosity for a pathogenic variant in *USH2A*. The *ABCA4* complex allele corresponded to a c.4926C>G transversion that predicts a p.Ser1642Arg missense mutation (Figures 4A1–A3) and c.5044_5058del that predicts an in-frame deletion of five amino acids (p.Val1682_Val1686del) in the ABCA4 protein (Figures 4B1–B3). The pathogenic variant identified in the *USH2A* gene was a c.2276G>T transversion that predicts an amino acid replacement of cysteine to phenylalanine at codon 759 of the protein (p.Cys759Phe) (Figures 4C1–C3). All three variants were present homozygously in DNA from the proband (Figures 4A3–C3), heterozygously in DNA from the proband’s father (Figures 4A2–C2), and were absent from maternal DNA. The pedigree and allele segregation is shown in Supplementary Figure S1. The *ABCA4* and *USH2A* variants have already been reported as pathogenic in a number of reports (Allikmets at al., 1997; Chacón-Camacho et al., 2013; Porto et al., 2017; Salles et al., 2017; DuPont et al., 2018; Pérez-Carro et al., 2018). NGS data files were analyzed for SNPs located at chromosome 1. A total of 24 SNPS occurring in 11 different genes, situated from 1p22.1 to 1q41 were identified. All 24 SNPs in proband’s DNA were present in the homozygous state, supporting the occurrence of paternal chromosome 1 UPD (Supplementary Table S1).

## Discussion

The frequency of UPD for any human chromosome has been estimated to be approximately 1 in 3,500 live births (Robinson, 2000). UPD can be classified according to parental origin or according to the size of the genomic region affected (Yamazawa et al., 2010). Two different processes may contribute to the UPD phenotypic effects and human diseases: the first of them involves a pathology if there is an underlying gene with genomic imprinting, while the second is related to the unmasking of a deleterious recessive gene with a pathogenic mutation, which in disomic chromosomes leads to homozygosity and consequently to recessively inherited diseases (Liehr, 2014).

In our case, concurrent homozygous pathogenic variants in both *ABCA4* and *USH2A* genes were identified in the proband, heterozygous in the paternal DNA, and absent in the maternal DNA. Additionally, SNP analysis indicated that 24 SNPs located in 11 loci along chromosome 1 were homozygous in the patient’s DNA. In conjunction, these data supported the occurrence of paternal UPD for chromosome 1 as the source of simultaneous homozygous mutations in *ABCA4* and *USH2A*. Various instances of recessive diseases associated with paternally derived UPD for chromosome 1 have been demonstrated, including infantile hypophosphatasia (*ALPl* gene) (Watanabe et al., 2014), neuronal ceroid lipofuscinosis-1 (*PPT1*) (Nilda et al., 2016; Travaglini et al., 2017), and atypical Hutchinson–Gilford progeria syndrome (*ZMPSTE24*) (Cassini et al., 2018) among others (Zeng et al., 2006; Miura et al., 2000; Manoli et al., 2010).

The simultaneous occurrence of *ABCA4* and *USH2A* recessive mutations in a single patient has not been previously reported and, to the best of our knowledge, only five affected patients with UPD for either *ABCA4* or *USH2A* gene have been described. In 2006, a 15-year-old female diagnosed with STGD was identified to carry a homozygous c.4139C>T (p.Pro1380Leu) *ABCA4* mutation. Parental DNA analyses showed that only her father was the carrier for the mutation, and genotyping of 24 short tandem repeats (STRs) on chromosome 1 demonstrated paternal UPD (Fingert et al., 2006). In 2007, a female with a clinical diagnosis of STGD was demonstrated to carry a homozygous c.3386G>T (p. Arg1129Leu) pathogenic variant in *ABCA4*, and analysis of microsatellite markers along the entire chromosome 1 showed that the isodisomy spanned a 4.4-Mb segment that included the *ABCA4* gene (Riveiro-Alvarez et al., 2007).

On the other hand, in a female patient with non-syndromic retinitis pigmentosa, a paternal UPD for the telomeric region of chromosome 1, including the *USH2A* gene, was demonstrated (Rivolta et al., 2002). In 2020, UPD for chromosome 1 was identified in a boy with non-syndromic hearing loss, and through STR analysis and homozygosity mapping, it was demonstrated that his homozygous *USH2A* variant arose from maternal UPD (Fu et al., 2020). Finally, a recent report described a case of paternal UPD resulting in homozygous mutations in both *USH2A* and *AGL* (glycogen storage disease type III) genes in a 4-year-old girl with congenital deafness, learning difficulties, and enlarged liver. No ocular anomalies were observed (Wang et al., 2021). This latter case is similar to our case since two pathogenic recessive mutations associated with two different diseases were unmasked by UPD for chromosome 1. However, our case is unique as chromosome 1 UPD resulted in homozygous pathogenic variants in two loci associated with IRD. Interestingly, but representing a disease mechanism other than uniparental isodisomy, a patient with compound heterozygous pathogenic variants in both *ABCA4* and *USH2A* genes has also recently been described (Liu et al., 2020). In the patient described by the authors, the clinical findings represented a combination of both retinal phenotypes.

The pathogenic variants identified in our patient have been previously described in the literature. The *ABCA4* c.4926C>G/c.5044_5058del variants have been demonstrated to occur in cis form, and thus, it is considered a complex *ABCA4* allele, as demonstrated in Brazilian STGD families (Porto et al., 2017; Salles et al., 2017). However, they can also occur as isolated pathogenic variants in individuals with STGD or cone dystrophy (Allikmets at al., 1997; Chacón-Camacho et al., 2013). c.2276G>T (p.759Phe) in *USH2A* is a common pathogenic variant identified in individuals with Usher syndrome type II or with isolated recessive retinitis pigmentosa (DuPont et al., 2018; Pérez-Carro et al., 2018). A previous study evaluated the ush2a ^p.(Cys771Phe)^ zebrafish model and identified the reduced usherin expression at the photoreceptor periciliary membrane, as well as increased rhodopsin levels in the photoreceptor cell body and decreased ERG b-wave amplitudes (Reurink et al., 2022).

Interestingly, no clinical evidence of Usher syndrome was observed in the retinal examination of our patient despite carrying a homozygous pathogenic mutation in *USH2A*. Mutations in *USH2A* are identified in 57–79% of cases of Usher syndrome (Koenekoop et al., 1999) but only in 10–15% of cases of non-syndromic retinitis pigmentosa (Bonnet et al., 2011). Although a formal hearing test was refused by the family, we found clinical evidence of hearing loss, which may be attributable to biallelic mutations in *USH2A*, whereas no retinitis pigmentosa features were observed in this girl; thus, it cannot be ruled out that a rod-cone dystrophy could develop at older age.

Finally, the recognition of biallelic pathogenic *USH2A* mutations in this STGD patient raises the need for frequent ophthalmological examinations for a potential early identification of retinitis pigmentosa manifestations.

## Data Availability

The datasets for this article are not publicly available due to concerns regarding participant/patient anonymity. Requests to access the datasets should be directed to the corresponding authors.
