# Supplementary material for: Case report: Disease phenotype associated with simultaneous biallelic mutations in ABCA4 and USH2A due to uniparental disomy of chromosome 1
Source: Front Genet. 2022 Aug 16;13:949437. doi: 10.3389/fgene.2022.949437 (PMC9424670; doi:10.3389/fgene.2022.949437)
Supplement: Supplementary file 1 [file DataSheet1.docx]

***Supplementary Material***

# Supplementary tables

| Gene | Locus | Variant | Genotype |
| --- | --- | --- | --- |
| *ABCA4* | 1p22.1 | c.6249C>T, p.Ile2083= | Homozygous |
| *ABCA4* | 1p22.1 | c.6282+7G>A, intronic | Homozygous |
| *ABCA4* | 1p22.1 | c.6285T>C, p.Asp2095= | Homozygous |
| *ABCA4* | 1p22.1 | c.6730-3T>C, intronic | Homozygous |
| *ABCA4* | 1p22.1 | c.6764G>T, p.Ser2255Ile | Homozygous |
| *COL11A1* | 1p21.1 | c.4609T>C, p.Ser1535Pro | Homozygous |
| *EMC1* | 1p36.13 | c.696G>A, p.Glu232= | Homozygous |
| *FLVCR1* | 1q32.3 | c.154G>C, p.Ala52Pro | Homozygous |
| *NEK2* | 1q32.3 | c.504T>C, p.His1680 | Homozygous |
| *NPHP4* | 1p36.31 | c.1470C>T, p.Leu490= | Homozygous |
| *NPHP4* | 1p36.31 | c.3570A>G, p.Glu1190Glu | Homozygous |
| *NPHP4* | 1p36.31 | c.86C>T, p.Thr29Met | Homozygous |
| *PEX10* | 1p36.32 | c.291A>G, p.Thr7= | Homozygous |
| *PEX14* | 1p36.22 | c.1032G>T | Homozygous |
| *PEX14* | 1p36.22 | c.84+8231A>G | Homozygous |
| *POMGNT1* | 1p34.1 | c.1414-16G>T, intronic | Homozygous |
| *SDCCAG8* | 1q43-q44 | c.1725G>A, p.Glu575= | Homozygous |
| *USH2A* | 1q41 | c.2256T>C, p.His752His | Homozygous |
| *USH2A* | 1q41 | c.10232A>C, p.Glu3411Gly | Homozygous |
| *USH2A* | 1q41 | c.12666A>G, p.Thr4222Thr | Homozygous |
| *USH2A* | 1q41 | c.373G>A, p.Ala125Thr | Homozygous |
| *USH2A* | 1q41 | c.4994T>C, p.Ile1665Thr | Homozygous |
| *USH2A* | 1q41 | c.504A>G, p.Thr168Thr | Homozygous |
| *USH2A* | 1q41 | c.6506T>C, p.Ile2169Thr | Homozygous |

**Table 1**. SNPs on chromosome 1.

# Supplementary Figures


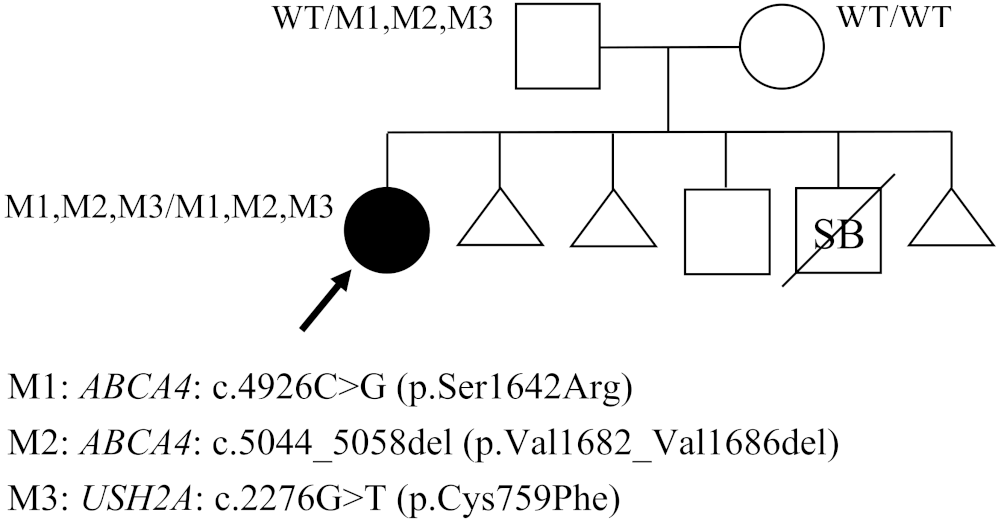


**Figure 1.** Pedigree structure and segregation analysis of mutations.
